# Successful Treatment of a Very Low-Birth-Weight Infant With GATA6 Neonatal Diabetes Using Continuous Subcutaneous Insulin Infusion

**DOI:** 10.7759/cureus.83555

**Published:** 2025-05-06

**Authors:** Takumi Ito, Atsushi Suzuki, Naoya Yamaguchi, Kohei Aoyama, Yutaka Negishi, Hajime Tanaka, Tohru Yorifuji, Atsushi Ishida

**Affiliations:** 1 Department of Pediatrics, Gifu Prefectural Tajimi Hospital, Tajimi, JPN; 2 Department of Pediatrics and Neonatology, Nagoya City University Graduate School of Medical Sciences, Nagoya, JPN; 3 Department of Pediatrics and Neonatology, Gifu Prefectural Tajimi Hospital, Tajimi, JPN; 4 Department of Pediatrics, Awano Children’s Clinic, Gifu, JPN; 5 Second Department of Internal Medicine, Japanese Red Cross Society Date Hospital, Date, JPN; 6 Division of Pediatric Endocrinology and Metabolism, Children’s Medical Center, Osaka City General Hospital, Osaka, JPN

**Keywords:** continuous glucose monitoring, continuous subcutaneous insulin infusion, gata6, neonatal diabetes mellitus, pancreatic aplasia, pancreatic exocrine insufficiency, very low birth weight infant

## Abstract

*GATA6* variants are associated with pancreatic hypoplasia/aplasia, congenital heart disease, and biliary tract disorders. We report the case of a very low birth weight infant (VLBWI) with pancreatic aplasia and neonatal diabetes caused by a previously reported *GATA6* variant. A male infant was born at 36 weeks and 0 days of gestation, weighing 1498 g, and presented with hyperglycemia on the first day of life. Continuous intravenous insulin was administered and discontinued after blood glucose levels normalized. Hyperglycemia recurred on day 7, necessitating insulin reinitiation with continuous glucose monitoring (CGM). Because of persistent glucose instability, the patient was transitioned to continuous subcutaneous insulin infusion (CSII). Improved glycemic control and reduced insulin dosage were achieved. Imaging failed to identify the pancreas, and serum trypsin levels were undetectable, confirming pancreatic aplasia. Poor weight gain owing to pancreatic exocrine insufficiency improved with pancreatic enzyme replacement; the resulting improvement in nutrient absorption necessitated an increase in insulin dosage. Genetic analysis revealed a heterozygous *GATA6* splice-site variant (c.1136-2A>G). CGM + CSII prevented ketoacidosis and severe hypoglycemia.

## Introduction

Neonatal diabetes mellitus (NDM) is rare, characterized by insulin secretion deficiency that develops before 6 months of age. The prevalence of NDM in Japan is estimated to be one in 89,000 live births [1]. NDM presents in two phenotypes: permanent NDM, which requires lifelong insulin therapy, and transient NDM, in which insulin can eventually be discontinued [2]. Insulin therapy is the primary treatment for NDM, including continuous intravenous infusion, subcutaneous pen-type injection, and continuous subcutaneous insulin infusion (CSII) using insulin pumps [3]. Regulating insulin dosage in low-birth weight infants is challenging, with difficulties in achieving good glycemic control. Although the efficacy of CSII in NDM has been reported [4-6], its application in very low birth weight infants (VLBWI) has rarely been documented.

Most NDM cases result from monogenic variants, with more than 20 causative genes having been reported [3]. One of these genes, *GATA6*, belongs to the GATA family of transcription factors (*GATA1-6*). *GATA4-6* is crucial for endodermal and mesodermal tissue development and differentiation [7]. *GATA6 *abnormalities (OMIM#600001) show autosomal dominant inheritance and are associated with pancreatic hypoplasia or aplasia, congenital heart defects, and biliary tract disorders. Reports of VLBWI with *GATA6 *gene abnormalities detailing the course of pancreatic enzyme replacement therapy (PERT) and CSII for pancreatic exocrine insufficiency are limited. We report a case of a VLBWI with congenital pancreatic aplasia, congenital heart disease, and biliary tract abnormalities associated with a previously reported *GATA6 *variant that achieved successful glycemic control with CSII.

This article was previously presented as a meeting abstract at the 58th Annual Congress of Japan Society of Perinatal and Neonatal Medicine on July 11, 2022. The parents provided written informed consent for genetic testing and the off-label use of continuous glucose monitoring (CGM) and agreed to share the patient’s clinical and genetic data.

## Case presentation

A male Japanese infant was born to a 28-year-old gravida one para 0 mother. The parents were not consanguineous. No family history of diabetes or heart disease was present. The mother was transferred to our hospital at 35 weeks and 6 days of gestation owing to fetal growth restriction. An emergency cesarean section was performed at 36 weeks and 0 days owing to fetal heart rate abnormalities. The Apgar scores were eight and nine at 1 and 5 min, respectively. The infant weighed 1498 g (z-score: -3.0), with a length of 43.3 cm (z-score: -1.2) and a head circumference of 31.7 cm (z-score: -0.3).

**Figure 1:**
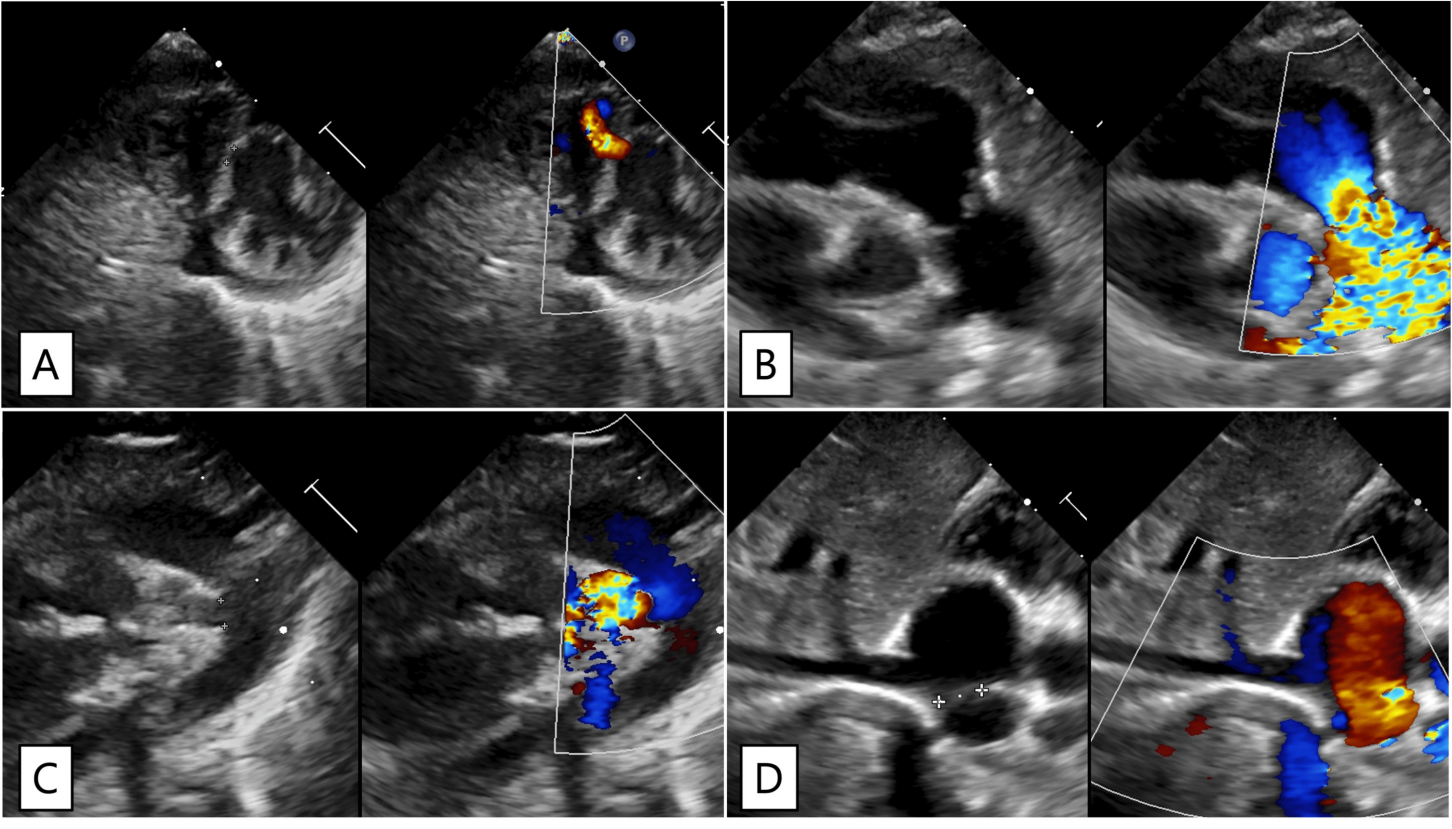
Echocardiographic findings. A: Muscular ventricular septal defect. Left-to-right shunting can be observed in the muscular portion of the interventricular septum; B: Valvular pulmonary stenosis. Post-stenotic doming of the pulmonary artery can be observed. The peak flow velocity in the main pulmonary artery is 3.6 m/s, corresponding to a pressure gradient of 52 mmHg; C: Patent ductus arteriosus. The diameter of the ductus arteriosus is 2.2 mm, with left-to-right shunting observed; D: Atrial septal defect (ASD). Left-to-right shunting can be observed at the atrial level. The size of the ASD was approximately 7 mm.

Initial laboratory tests revealed a blood glucose level of 49 mg/dL (institutional reference range: 70-110 mg/dL); ultrasound showed a muscular ventricular septal defect, valvular pulmonary stenosis, patent ductus arteriosus, and an atrial septal defect (Figure 1).

**Figure 2:**
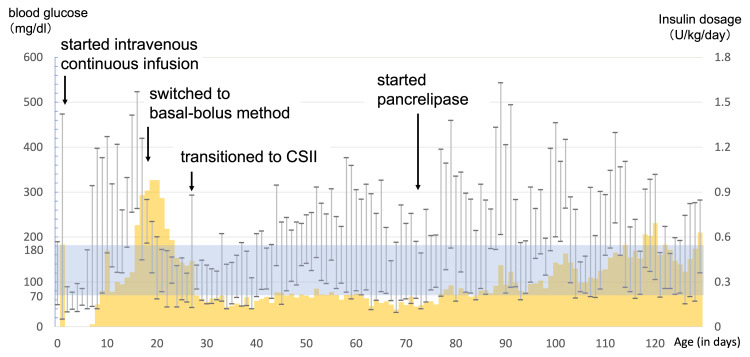
Blood glucose and insulin dosage during hospitalization. The yellow bars indicate the daily insulin dosage, whereas the vertical range lines indicate the range of blood glucose fluctuation (minimum to maximum values per day). The target range for blood glucose levels (70–180 mg/dL) is color-coded.

A 10% glucose solution was administered intravenously (infusion rate: 3.9 mg/kg/min). Hyperglycemia (455 mg/dL; reference: 70-110 mg/dL) was observed on day 1. With infection ruled out and blood insulin levels remaining undetectable, continuous regular intravenous insulin was initiated; blood glucose levels normalized immediately, and insulin was discontinued on the same day. Although blood glucose levels remained normal during the week, hyperglycemia (up to 314 mg/dL) requiring intravenous insulin resumption relapsed on day 7. Considering the recurrent undetectable blood insulin levels, NDM was suspected. CGM using the FreeStyle Libre® system (Abbott, Abbott Park, Illinois, USA) was initiated alongside regular intravenous insulin, with blood glucose measured several times a day. Although initial levels were managed, glycemic control remained unstable. On day 15, treatment was adjusted by adding postprandial intravenous insulin boluses (0.01-0.06 U/kg) to the fixed-rate insulin infusion (0.005-0.02 U/kg/h). Blood glucose gradually stabilized, and the total insulin requirement decreased, though it could not be discontinued. On day 27 (body weight: 1,824 g), treatment was transitioned to CSII using the MiniMed 640G® system (Medtronic, Minneapolis, Minnesota, USA), with insulin diluted 10-fold with normal saline. Plasma and urinary 24-h C-peptide levels on day 34 were <0.01 ng/mL and 0.02 μg/day, respectively. The initial CSII regimen consisted of basal insulin at 0.005 U/h and bolus insulin doses based on preprandial blood glucose levels: 0.01 U for <50 mg/dL, 0.02 U for 50-99 mg/dL, and 0.04 U for 100-199 mg/dL, with incremental increases of 0.02 U per 100 mg/dL increase in blood glucose. Figure 2 shows the trends in blood glucose and insulin doses.

However, poor weight gain (<10 g/day) persisted despite appropriate insulin therapy and full enteral nutrition. Given abnormalities suggestive of hepatobiliary dysfunction and associated malabsorption, including markedly elevated gamma-glutamyl transpeptidase (γ‐GTP) (>1000 U/L; reference: 13-64 U/L), a declining prothrombin time (30-50%; reference: 80-150%) from birth, persistently elevated total bile acids, and a gradual increase in direct bilirubin to approximately 1.8 mg/dL (reference: 0.2-0.8 mg/dL), medium-chain triglyceride milk was administered from day 37. However, it did not result in adequate weight gain.

**Figure 3:**
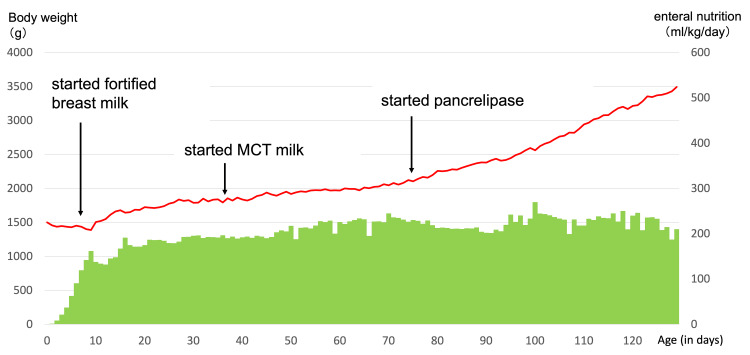
Enteral nutritional intake and body weight during hospitalization. The bar graph indicates the change in daily enteral nutrition, and the line graph indicates the change in body weight. Weight gain improved after pancrelipase administration.

Ultrasonography failed to reveal the pancreas, and trypsin was undetectable in blood tests on day 63. Sudan Ⅲ staining of fecal fat was positive, and fecal elastase was below the detection limit (<200 μg/g). Based on these findings, pancreatic aplasia with pancreatic exocrine insufficiency was diagnosed. PERT with pancrelipase (60 mg/kg/day in eight doses) was initiated on day 73, which improved weight gain. Although milk intake per body weight remained unchanged, insulin requirements per body weight gradually increased (Figures 2-3).

**Figure 4:**
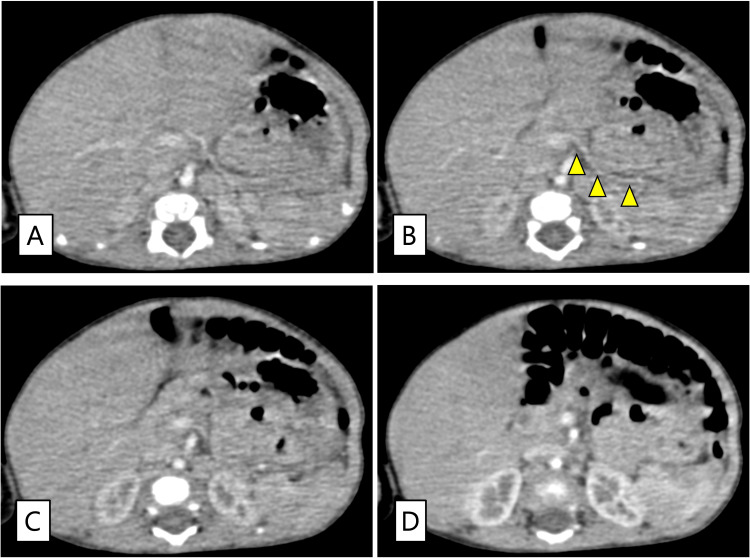
Abdominal contrast-enhanced computed tomography (CT). The images are 1-mm slice CT scans arranged in cranial-to-caudal order from panels A to D. The yellow arrowhead in panel B indicates the splenic artery. No pancreatic tissue can be observed ventral to the splenic vessels. The gallbladder is absent in the caudal portion of the right hepatic lobe, where it is typically located.

Subsequently, basal and bolus insulin doses were adjusted according to blood glucose levels, body weight, and the amount of milk feeding. From day 112, basal insulin was fixed at 0.025 U/h, with adjustable bolus doses. Carbohydrate counting was performed on day 125, increasing insulin levels by 0.025 U per 30 mL of milk. The patient was discharged on day 130 with stable glycemic control on CSII, without any severe hypoglycemic episodes during hospitalization. The mean absolute relative difference (MARD), calculated from the simultaneous evaluation of CGM values and blood glucose measurements during hospitalization, was 17.8% based on an analysis using 414 sample pairs. Contrast-enhanced computed tomography at 5 months of age also failed to definitively identify the pancreas or gallbladder (Figure 4).

Insulin dilution was discontinued at 5 months of age. At 5 years of age, the patient was treated with CSII, CGM, and PERT; his psychomotor levels and developmental milestones were age-appropriate.

**Figure 5:**
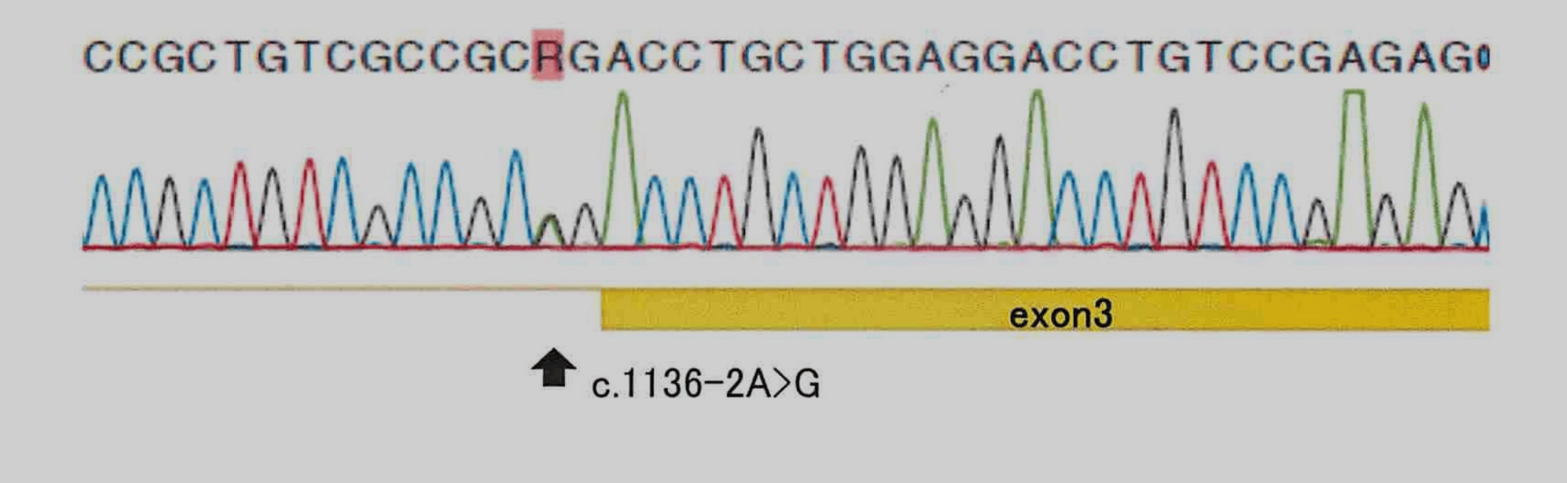
Sanger sequencing of all exons of the GATA6 gene. A heterozygous c.1136-2A>G splice variant was identified. DNA was extracted using the QIAamp DNA Mini Kit. PCR was performed with 50 ng of DNA per reaction under the following conditions: 94°C for 30 s, 60°C for 30 s, and 72°C for 1 min, for 30 cycles. Primers (20–25 mer) were designed based on the NCBI reference sequence to amplify all exons and exon-intron boundaries. Sequencing was conducted using the BigDye Terminator v3.1 Cycle Sequencing Kit, and data were analyzed with Mutation Surveyor version 3.25.

Considering the pancreatic aplasia and heart defects, *GATA4 *and *GATA6 *were analyzed after obtaining written informed consent for genetic testing from the patient’s parents. Sanger sequencing identified a previously reported heterozygous splice-site variant in *GATA6 *(c.1136-2A>G) (Figure 5) [8].

## Discussion

Insulin therapy for NDM remains challenging. Neonates require frequent feeding in variable amounts; they have a small but rapidly increasing body weight with significant blood glucose fluctuations, making recognizing hypoglycemic symptoms difficult. In this case, glycemic control could not be achieved with fixed-rate continuous intravenous insulin infusion, though the addition of postprandial intravenous boluses subsequently improved glycemic control. However, a change in insulin administration was necessary to anticipate post-discharge care. Several reports have suggested that combining CSII with CGM is beneficial for the management of neonatal diabetes [9-11]. In this case, CGM-assisted CSII was deemed desirable owing to significant blood glucose fluctuations and the complexity of frequent blood sampling and injections. For CGM insertion, we selected the site with the thickest subcutaneous tissue, measured by ultrasound, and gathered the skin to avoid the needle tip making contact with muscle or bone during puncture. CSII implementation in this case required adaptations to the insertion method and insulin dilution. In previous reports, the smallest infant treated with CSII was born at 730 g, and CSII was initiated when the infant reached 750 g [12]. In that case, the MiniMed™ Silhouette™ infusion set catheter was modified to be shorter, whereas in our case, the catheter was inserted manually without modification. Owing to the minimum flow rate constraints of the pump, insulin was diluted 10-fold to achieve more precise dosing. Transient hyperglycemic episodes occurred after the introduction of CGM + CSII, but the patient did not develop ketoacidosis or severe hypoglycemia. Insulin dilution enabled a more flexible dose adjustment, showing that CGM + CSII-based blood glucose management can be beneficial in VLBWI. However, the accuracy of CGM in the neonatal period remains a topic of discussion.

Reports on the accuracy of FreeStyle Libre® in neonates are limited, and studies specifically evaluating its accuracy in NDM are scarce. A study evaluating its accuracy in terms of neonates without complications reported MARD values ranging from 15.8% to 18.9% [13], whereas another study evaluating its accuracy in children with type 1 diabetes reported MARD values between 10.4% and 24.7% [14]. The MARD observed in the present case was comparable to these values but not sufficient to be considered highly accurate. However, it may have contributed to an approximate understanding of blood glucose levels and a reduction in the frequency of measurements.

The primary mechanisms of NDM are pancreatic malformations or β-cell dysfunction. Recent advances in molecular genetics have revealed that most cases result from single-gene variants that often cause complications beyond diabetes [15]. Gene variants affecting pancreatic morphology, such as *GATA6 *in this case, are rare. *GATA6 *variants are associated with cardiac malformations, pancreatic hypoplasia, endocrine disorders (such as pituitary hypoplasia and hypothyroidism), and gastrointestinal abnormalities (including intestinal malrotation, micro-colon, gallbladder hypoplasia, biliary atresia, and rectal bleeding) [16]. Intrauterine insulin deficiency caused by pancreatic hypoplasia often results in intrauterine growth restriction and low birth weight. In this case, the diagnosis was prompted by pancreatic aplasia, congenital heart disease, and gallbladder hypoplasia. When pancreatic aplasia/hypoplasia is accompanied by heart disease, *GATA6 *variants should be considered. NDM with *GATA6 *variants may require treatment for exocrine insufficiency and glycemic control owing to pancreatic aplasia/hypoplasia. Pancreatic exocrine insufficiency causes growth impairment through nutrient malabsorption, resulting in reduced calorie intake from fat maldigestion and malabsorption of fat-soluble vitamins [17]. PERT without fat restriction is commonly indicated for pancreatic exocrine dysfunction [18]. In this case, PERT might have improved malabsorption, leading to an increase in insulin requirements per body weight, showing that PERT could improve weight gain in neonates with pancreatic exocrine insufficiency. In *GATA6*-related NDM with pancreatic exocrine insufficiency, insulin deficiency and pancreatic exocrine insufficiency contribute to impaired weight gain. Therefore, careful attention should be given to the increase in insulin requirements following the initiation of PERT. The limitation of this study is that it was difficult to generalize the findings, as this is a single case report, and *GATA6 *variation causes a wide spectrum of symptoms.

## Conclusions

CSII combined with CGM has been found to be a useful therapeutic option even in VLBWI, although precautions such as manual catheter insertion, careful selection of puncture sites, and dilution of the insulin solution are necessary. NDM associated with *GATA6 *variants is caused by pancreatic hypoplasia/aplasia and may also require PERT as a result of exocrine pancreatic insufficiency. Importantly, from the perspective of neonatal nutrition, the combination of CSII and PERT plays a crucial role in managing neonatal *GATA6 *diabetes complicated by exocrine pancreatic insufficiency.
